# Hide-and-Seek With Spindle-Cell-Component-Poor Metaplastic Thymoma

**DOI:** 10.7759/cureus.60136

**Published:** 2024-05-12

**Authors:** Gintarė Ražanskienė, Vilhelmas Landsbergis, Vytenis Bertašius, Rimvydas Norvilas, Ugnius Mickys

**Affiliations:** 1 National Center of Pathology, Vilnius University Hospital Santaros Klinikos, Vilnius, LTU; 2 Faculty of Medicine, Vilnius University, Vilnius, LTU; 3 Thoracic Surgery, Vilnius University Hospital Santaros Klinikos, Vilnius, LTU; 4 Hematology, Oncology and Transfusion Medicine Center, Vilnius University Hospital Santaros Klinikos, Vilnius, LTU

**Keywords:** islet-1, paraneoplastic syndrome, psoriasis, yap1::maml2, metaplastic thymoma

## Abstract

Metaplastic thymoma is a rare biphasic thymic tumor with indolent behavior and recurrent *YAP1::MAML2* gene rearrangement. Although the diagnosis of this tumor is usually straightforward based on hematoxylin and eosin (H&E) findings alone, cases with scant spindle-cell (“pseudosarcomatous stroma“) components can be easily confused with more commonly occurring type A thymoma. We present a case of metaplastic thymoma with a sparse stroma-like spindle-cell component, discussing its histological and immunohistochemical hints and drawing attention to the visual similarity to type A thymoma. This is also the first published case of metaplastic thymoma with associated psoriasis.

## Introduction

Suster et al. first described the tumor, that we now call “metaplastic thymoma” in 1997, naming it “thymoma with pseudosarcomatous stroma” and stressing its biphasic pattern with peculiar cellular “stroma” [1]. While Suster et al. considered a stroma-like component to be reactive in nature, a subsequent study by Yoneda et al. [2] argued it to be a mesenchymal metaplasia of tumor cells, i.e., derived from the same neoplastic precursor as the epithelioid component. Nonetheless, Yoneda et al. called the tumor a “low-grade metaplastic carcinoma of the thymus,” on the ground of the slight to moderate cytologic atypia and the absence of organotypic features such as lobulated architecture, perivascular spaces, and intratumoral thymocytes [2]. In 2004, the WHO included the tumor in the new classification of thymic tumors under the name “metaplastic thymoma,” emphasizing its indolent behavior. We describe a case of metaplastic thymoma with scant stroma-like spindle-cell component. We discuss “pearls and pitfalls” differentiating in such scenarios with type A thymoma. This case is also the first published case of metaplastic thymoma with associated psoriasis.

## Case presentation

A 32-year-old male patient was referred to our hospital by a family doctor. He complained of nonpersistent heart palpitations and episodic shortness of breath. Prior medical history was unremarkable except for a compound odontoma, diagnosed 10 years ago, and scalp psoriasis, presented and diagnosed by a dermatologist eight years ago. He had no familial history of psoriasis or other skin diseases.

**Figure 1:**
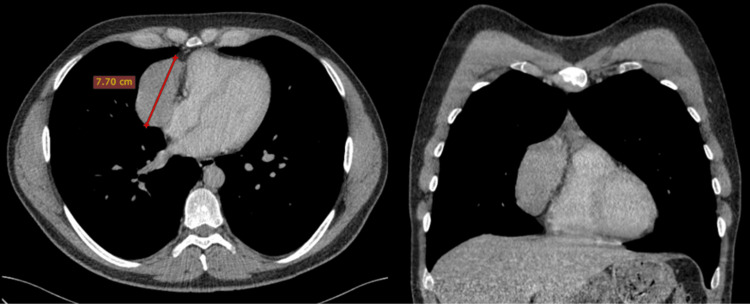
Axial and coronal contrast-enhanced CT CT: Computed tomography Axial and coronal contrast-enhanced CT: Well-defined hypovascular homogeneously enhancing anterior mediastinum mass along the right border of the heart.

Chest radiograph revealed widening of the anterior mediastinum, and subsequent contrast-enhanced computed tomography (CT) scan showed a well-defined hypovascular homogeneously enhancing anterior mediastinum mass along the right border of the heart measuring over seven centimeters (Figure 1).

**Figure 2:**
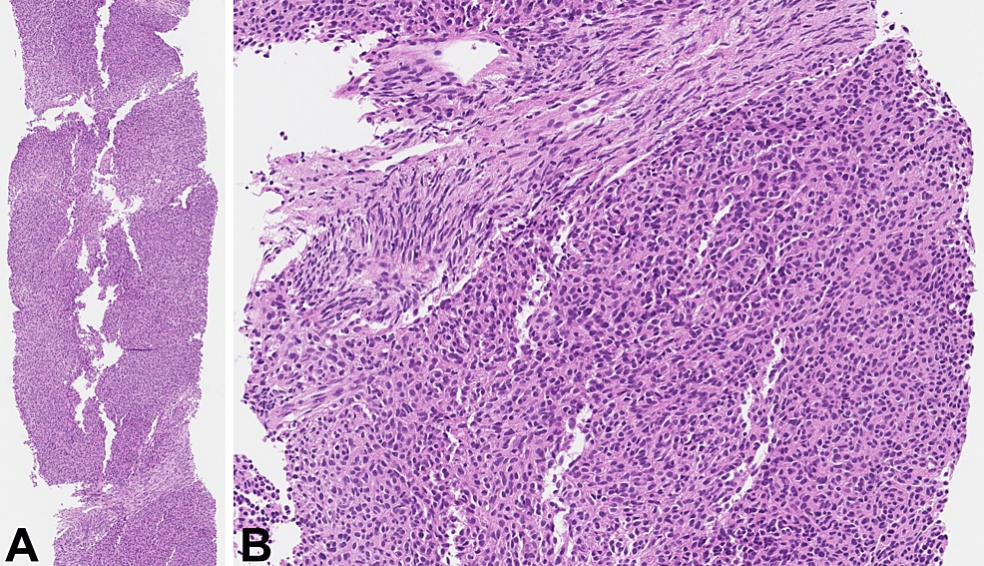
Biopsy core of the mass H&E: Hematoxylin and eosin The tumor demonstrates solid areas of epithelioid cells (A, H&E 40x). Intersecting the solid areas is a minor stroma-like component with densely arranged fibroblast-like cells (B, H&E 200x).

A tru-cut core biopsy of the lesion was performed. Histologically, the tumor was mainly composed of solid sheets of medium-sized epithelioid cells with eosinophilic cytoplasm and oval brand nuclei without conspicuous nucleoli. Only a couple of intervening stroma-like bands were evident, composed of densely arranged cells with elongated, slightly wavy nuclei (Figure 2).

**Figure 3:**
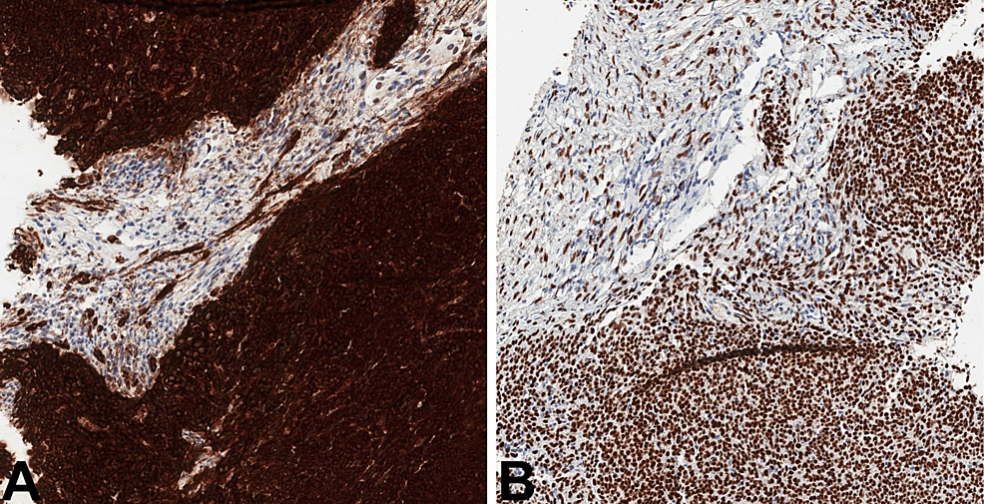
Immunohistochemical features of the tumor Keratin AE1/AE3: Strong diffuse reaction in solid areas and focal weak staining in stroma-like component (A, 100x). Islet-1: Strong nuclear reaction in solid areas with oval nuclei as well as in stroma-like component with elongated, thin, wavy nuclei (B, 100x).

The tumor showed diffuse strong reaction for keratin AE1/AE3, Pax8, and Islet-1. Importantly, not only solid sheets of epithelioid cells showed positivity for keratin AE1/AE3 and Islet-1, focal positivity was evident in the minor stroma-like component, one of the key features that was primarily overlooked (Figure 3).

Tumor cells were negative for CD20, CD5, Sox-10, desmin, and α-smooth muscle actin. Only a couple of scattered lymphoid cells were terminal deoxynucleotidyl transferase (TdT) positive. The conclusion of the biopsy findings at that time was thymoma type A. 

**Figure 4:**
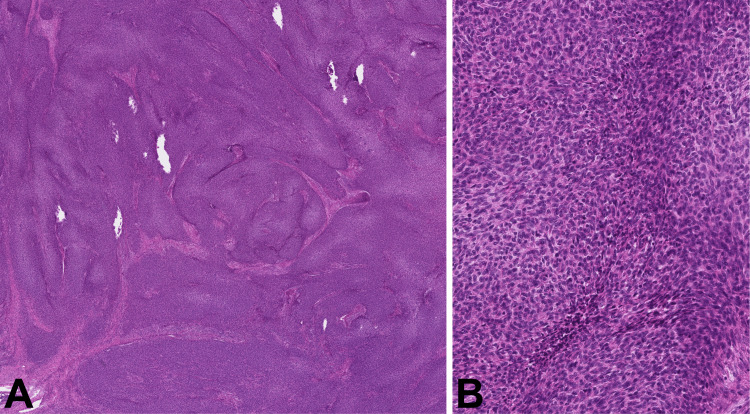
Histological pattern and cytology of the tumor H&E: Hematoxylin and eosin Fusing ribbonlike structures (A, H&E 20x). Cytology of the predominant component: short bland spindle cells with different shades of cytoplasm, giving a “3D impression“ (B, H&E 200x).

The patient underwent surgery, using a Cambridge Medical Robotics (CMR) Versius robotic system through the three ports accessing the right pleural cavity. The tumor was fragmented during the extraction from the pleural cavity. The postoperative period was uneventful. Grossly, the tumor appeared as a firm, well-circumscribed, encapsulated mass with grey-to-yellow cut surface. On microscopic evaluation, the tumor showed fussing ribbonlike structures, composed of bland epithelioid or short spindle cells (thymoma type A-like) with darker or focally lighter eosinophilic cytoplasm, giving a “3D impression“ (Figure 4).

**Figure 5:**
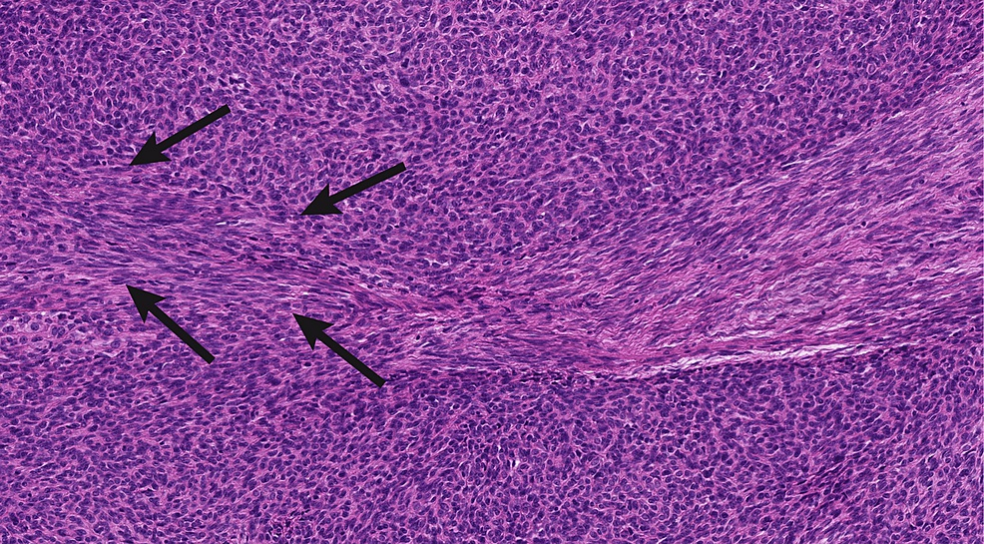
Interface between the two components of the tumor H&E: Hematoxylin and eosin Gradual transition (arrows) between the two components: thymoma type A-like component and stroma-like component with spindle cells resembling fibroblasts (H&E x200).

Accompanying these bands was a minor component, resembling odd-looking stroma with densely arranged, elongated, narrow nuclei and thin wavy collagen fibrils. The gradual transition between the two components was focally evident (Figure 5).

**Figure 6:**
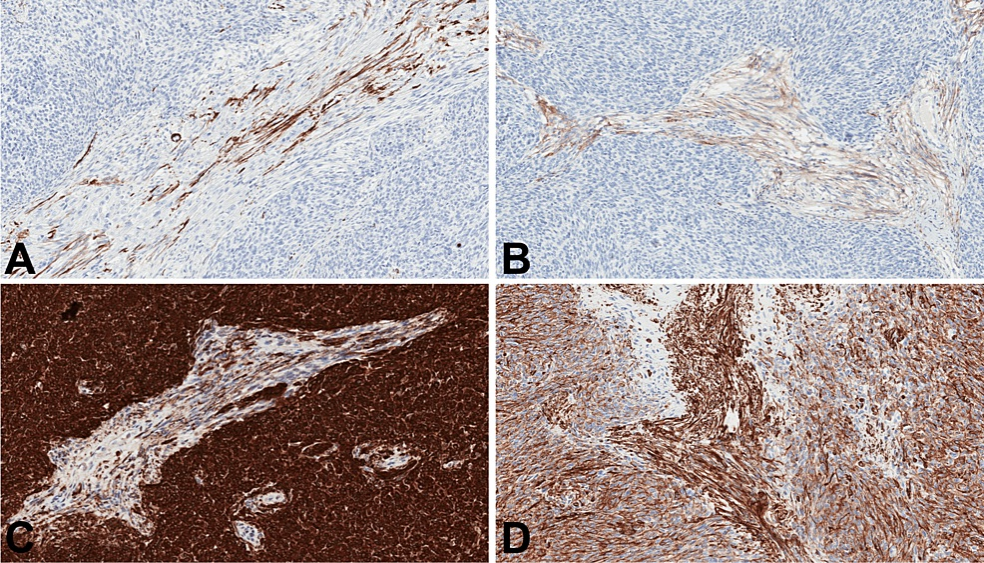
Immunophenotype of the resected tumor Immunostaining for α-smooth muscle actin focally positive in the stroma-like component and negative in the predominant component (A, 100x). Likewise, focal positivity for EMA in the stroma-like component and negative reaction in the predominant component (B, 100x). Same as in the biopsy, reaction for keratin AE1/AE3 was diffuse and strong in the predominant component and focal/weak in the stroma-like component (C, 100x). Reaction for vimentin was diffuse in both of the components, being slightly more intense in the stroma-like component (D, 100x).

There were no necrosis or mitotic activity. Component of short spindle cells demonstrated diffuse strong positivity for keratin AE1/ E3, keratin 5/6, p63, and vimentin. It showed no expression of cluster of differentiate 20 (CD20), epithelial membrane antigen (EMA) and α-smooth muscle actin. Stroma-like component showed strong positivity for vimentin, faint positivity for EMA, as well as focal positivity for α-smooth muscle actin and keratin AE1/AE3 (Figure 6).

TdT-positive thymocytes were absent, except for small areas of the residual thymic tissue at the periphery of the tumor. Ki67 proliferation index was only up to 2%. Finally, to prove the diagnosis of metaplastic thymoma, we performed targeted RNA sequencing, using TruSight RNA Pan-Cancer gene panel (Illumina, San Diego, CA, USA). Sequencing data analysis revealed an in-frame fusion between exon 1 of the YAP1 gene and exon 2 of the MAML2 gene on chromosome 11. The postoperative period was uneventful. Interestingly, during his first follow-up a month after the operation, the patient stressed that his psoriatic lesions of the scalp had cleared up completely without any specific treatment.

## Discussion

The proportion of stroma-like components in metaplastic thymoma may vary significantly [3]. Our case represents the spindle-cell-poor end of the spectrum, making it quite challenging to come up with the right diagnosis. The main diagnostic pitfall in this scenario would be the type A thymoma. The major hint leading to the correct diagnosis should be the unusual “stroma” with dense fibroblast-like cells, regardless of its proportion in the tumor. Another histological difference between the two types is the scattered thymocytes in type A thymoma which is typically lacking in the metaplastic type [4]. An unusual immunomarker expression in the stroma-like component, such as EMA, keratin, and Islet-1 could also be helpful. Islet-1 is normally expressed in the thymic epithelium as well as in thymic epithelial tumors (TET)[5]; therefore, Islet-1 expression in the stroma-like component supports its epithelial nature.

A number of recurrent molecular aberrations in TET have already been identified, making molecular studies for TET a diagnostically important tool. Intriguingly, most of the recurrent gene fusions in TET involve mastermind-like transcriptional coactivator 2 (MAML2) gene, namely, MAML2::CRTC1 in mucoepidermoid carcinoma, MAML2::KMT2A in a subset of B2 and B3 thymomas, and importantly regarding the current case, YAP1::MAML2 in metaplastic thymoma [4]. Zhao et al. in their study of 17 metaplastic thymoma cases proved that YAP1::MAML2 rearrangement is found in both epithelioid and spindle cell components, which again confirms the neoplastic origin of the spindle cell/stroma-like component [6].

Paraneoplastic psoriasis has only been described in a couple of cases regarding thymic tumors [7-8]. The prototypical paraneoplastic syndrome associated with thymoma is myasthenia gravis (MG), with occurrence of 30%-50% in thymoma patients generally [9]. Yet, only few reports of metaplastic thymoma with MG have been published [10,11]. MG and other thymic tumor-related paraneoplastic syndromes are thought to be triggered by autoreactive CD4+ T and a reduced level of regulatory T cells, as a consequence of disordered thymopoiesis inside the tumor [12]. Findings of the earlier mentioned that MG associated metaplastic thymoma cases are conflicting, while one of the cases demonstrated the usual thymocyte-depleted histology [10], and the other case exhibited unusual intratumoral TdT+ lymphocytic accumulation [11]. As well as the former, our case showed TdT-positive lymphocytes only in the residual thymic tissue; hence, there was no thymopoiesis inside the tumor.

## Conclusions

This case demonstrates a rare histological presentation of metaplastic thymoma with scant stroma-like component, resembling a more commonly encountered type A thymoma. Regardless of the minute amount, the unusually high cellularity with fibroblast-like cytology of the odd-looking “stroma” and a specific immunophenotype should hint at this rare type of thymoma.
